# Facile Synthesis of ZnO Nanoparticles on Nitrogen-Doped Carbon Nanotubes as High-Performance Anode Material for Lithium-Ion Batteries

**DOI:** 10.3390/ma10101102

**Published:** 2017-09-21

**Authors:** Haipeng Li, Zhengjun Liu, Shuang Yang, Yan Zhao, Yuting Feng, Zhumabay Bakenov, Chengwei Zhang, Fuxing Yin

**Affiliations:** 1 School of Materials Science & Engineering, Research Institute for Energy Equipment Materials, Tianjin Key Laboratory of Materials Laminating Fabrication and Interface Control Technology, Hebei University of Technology, Tianjin 300130, China; lihp_hebut@outlook.com (H.L.); lzj125@126.com (Z.L.); hebutyangshuang@163.com (S.Y.); yinfuxing@hebut.edu.cn (F.Y.); 2 Synergy Innovation Institute of GDUT, Heyuan 517000, China; 18222385469@163.com; 3 Institute of Batteries LLC, Nazarbayev University, 53 Kabanbay Batyr Avenue, Astana 010000, Kazakhstan; zbakenov@nu.edu.kz

**Keywords:** lithium ion battery, anode, ZnO/nitrogen-doped carbon nanotube (ZnO/NCNT) composite, highly-dispersed ZnO nanoparticles, sol-gel

## Abstract

ZnO/nitrogen-doped carbon nanotube (ZnO/NCNT) composite, prepared though a simple one-step sol-gel synthetic technique, has been explored for the first time as an anode material. The as-prepared ZnO/NCNT nanocomposite preserves a good dispersity and homogeneity of the ZnO nanoparticles (~6 nm) which deposited on the surface of NCNT. Transmission electron microscopy (TEM) reveals the formation of ZnO nanoparticles with an average size of 6 nm homogeneously deposited on the surface of NCNT. ZnO/NCNT composite, when evaluated as an anode for lithium-ion batteries (LIBs), exhibits remarkably enhanced cycling ability and rate capability compared with the ZnO/CNT counterpart. A relatively large reversible capacity of 1013 mAh·g^−1^ is manifested at the second cycle and a capacity of 664 mAh·g^−1^ is retained after 100 cycles. Furthermore, the ZnO/NCNT system displays a reversible capacity of 308 mAh·g^−1^ even at a high current density of 1600 mA·g^−1^. These electrochemical performance enhancements are ascribed to the reinforced accumulative effects of the well-dispersed ZnO nanoparticles and doping nitrogen atoms, which can not only suppress the volumetric expansion of ZnO nanoparticles during the cycling performance but also provide a highly conductive NCNT network for ZnO anode.

## 1. Introduction

Lithium-ion batteries (LIBs) have been widely used as energy sources because of their high energy density and excellent recycling time [1,2,3,4]. In recent years, graphite has become the main commercial anode material for LIBs. However, its relatively low capacity 372 mAh·g^−1^ (theoretical value) limits further development of LIB [5]. Significant attention has been focused on the research of next-generation promising anode candidates to replace graphite. Transition metal oxides (MO_x_, where M is Fe, Co, Ni, Cu, Zn, etc.) have been considered as new type alternative anode materials for next-generation high-capacity LIB due to their high theoretical capacity [6]. Particularly, nontoxic zinc oxide (ZnO) has received considerable attention owing to its outstanding advantages, such as high theoretical capacity (978 mAh·g^−1^), nontoxicity, low processing cost, and chemical stability. Despite of these merits, the potential application of ZnO anode is limited by the sever capacity fading, poor cycling stability, and high irreversible capacity, which are caused by its agglomeration/pulverization and large volume expansion/contraction during the cycling process [7,8,9]. Moreover, its unsatisfactory electrical conductivity suppresses fast lithium diffusion, leading to poor lithium storage behavior [10].

Up to now, many strategies have been researched to overcome mentioned drawbacks of ZnO based anodes. One of the strategies is to utilize nanostructure features in the material, which can improve mechanical durability of ZnO and shorten the diffusion paths for Li ion transport, thereby improving cycling performance and facilitating fast lithium-ion diffusion [7,11]. Nevertheless, nanostructured ZnO with the high surface area favors side reaction with the electrolyte, which leads to irreversible capacity loss during the initial cycle. Furthermore, this approach is insufficient to ameliorate the poor electric conductivity of ZnO sufficiently [12,13]. In order to further solve the shortcomings of the ZnO anodes, nanostructured ZnO could be combined with a highly conductive and elastic matrix to form a nanocomposite structure. In the past few years, various hybrid nanostructures of ZnO have been developed, such as carbon/ZnO nanorods [14], ZnO/mesoporous carbon [15], ZnO@Graphene [16], and ZnO/carbon nanotube (ZnO/CNT) [7]. Ren et al. reported that the carbon-encapsulated ZnO tetrahedron were synthesized via a simple internal-reflux approach, and this composite anode demonstrated a desirable capacity of 518 mAh·g^−1^ at a current density of 110.7 mA·g^−1^ [17]. Guler et al. reported that the ZnO/CNT nanocomposites were prepared via thermal evaporation techniques followed by an in situ plasma oxidation, and the reversible capacities could be retained as 527 mAh·g^−1^, even after 100 cycles [18]. Among the other conducting agents, CNT has been considered to be a very promising one, which could form a conductive network and buffer the volume expansion of ZnO upon cycling [19,20,21]. However, due to the inert nature of CNT, it is difficult to obtain highly homogeneously dispersed metal oxide composite via common techniques. Recently, Qin et al. [22,23,24] reported that nitrogen doping of CNT could improve its surface activity and provide more nucleation sites to disperse metal oxide on its surface, which facilitates the morphology and particle size control of the hybrids. Specifically, nitrogen doping is considered as a feasible way to promote effectively the conductivity and reactivity of carbonaceous materials, which will increase electrochemical properties and reversible capacity of the hybrids, as has been experimentally and theoretically studied [25,26,27]. However, to our knowledge, only a few studies have been reported to date on synthesis and electrochemical properties of ZnO nanoparticles deposited on nitrogen-doped carbon nanotube (NCNT).

Herein, we report on a simple environmentally friendly sol-gel method for homogeneous deposition of ZnO nanoparticles on the NCNT surface, and investigation of physical and electrochemical performance of the resulted ZnO/NCNT composite as an anode for laboratory scale lithium-ion cells.

## 2. Materials and Methods 

### 2.1. Chemical Materials

Zinc acetate (Zn(CH_3_COO)_2_, ≥99%, Tianjin Fuchen Chemical Reagents Factor), lithium hydroxide (LiOH, ≥90%, Tianjin Guangfu Fine Chemical Research Institution) and ethanol (C_2_H_5_OH, ≥99.7%, Tianjin Fuchen Chemical Reagents Factor) were used as metal-ion source, catalyst, and solvent, respectively. Carbon nanotubes (CNT, Tianjin Guangfu Fine Chemical Research Institution) and nitrogen-doped carbon nanotubes (NCNT, N content 2.98%, Tianjin Guangfu Fine Chemical Research Institution) were used as a conductive supportive matrix.

### 2.2. Preparation of ZnO/NCNT and ZnO/CNT

The synthetic procedures for ZnO/NCNT and ZnO/CNT were adopted from the literature [28]. The ZnO/NCNT nanocomposite was fabricated by the sol-gel route. Briefly, Zn(CH_3_COO)_2_ and LiOH (molar ratio = 1.3:1) were dissolved separately in 130 dm^3^ ethanol with magnetic agitation at room temperature until the reagents were completely dissolved. The Zn(CH_3_COO)_2_-LiOH mixed system was formed by adding the LiOH solution to the Zn(CH_3_COO)_2_ solution dropwise under agitation on a magnetic stirrer for 30 min at room temperature. Subsequently, 0.085 g NCNT was added to the mixture solution. This resulted in formation of black sol, which was continued stirring for 24 h. The black sediment of ZnO/NCNT was obtained through filtering separation, alternately cleaned with distilled water and ethanol for several times. Then, it was dried in vacuum at 60 °C for 12 h. The preparation of ZnO/CNT was same as described for ZnO/NCNT with substitution of CNT for NCNT.

### 2.3. Material Characterizations

The samples crystalline structure was examined by X-ray diffraction (XRD) on a Rigaku Corporation Smartlab system (Tokyo, Japan) with Cu-Kα radiation (λ = 0.15418 nm) source, and their morphology and structure were investigated by means of transmission electron microscopy (TEM, JEM-2100F, JEOL, Tokyo, Japan) and scanning electron microscopy (SEM, Hitachi Limited S-4800, Tokyo, Japan). Surface elemental analysis was carried out by an energy dispersive X-ray spectroscopy (EDX) attached to the SEM apparatus. X-ray photoelectron spectroscopy (XPS) measurements of the samples were recorded on a Thermo K-Aepna Ultra spectrometer (Waltham, MA, USA) with an Mg-Kα excitation source. Thermo-gravimetric (TG, SDT Q-600, TA Instruments-Waters LLC, Newcastle, PA, USA) analysis was conducted from room temperature to 1000 °C under air with a heating rate of 10 °C min^−1^.

### 2.4. Electrochemical Measurements

A two-electrode CR2025 coin cells were assembled in a glovebox (MBraun) filled with argon and employed to assess the electrochemical performance of the samples. The working electrodes were obtained by a slurry coating process. The slurries were formed by mixing active materials (ZnO/NCNT or ZnO/CNT), carbon black (CB), and polyvinylidene fluoride (PVDF) with a mass ratio of 80:10:10 in *N*-methyl-2-pyrrolidine (NMP). The resulting homogenously slurries were coated on copper foil which served as current collector and dried at 60 °C under a vacuum oven for 12 h to remove solvent and water. Then the working electrodes were cut with 1.5 cm diameter and a mass loading of ~0.85 mg cm^−2^ as the anode. The pure lithium metal foil was used as reference electrode. The polypropylene micro-porous film (Cellgard 2300) was used as a separator. The electrolyte was 1.0 M LiPF_6_ dissolved in a mixture of ethylene carbonate/diethylcarbonate/dimethylcarbonate (EC/DEC/DMC) (1:1:1 by volume). Galvanostatic charge–discharge tests were detected on NEWARE battery test system (Shenzhen, China) with a potential range from 0.005 and 3.0 V vs. Li/Li^+^, at a wide range of current rates.

## 3. Results and Discussion

**Figure 1:**
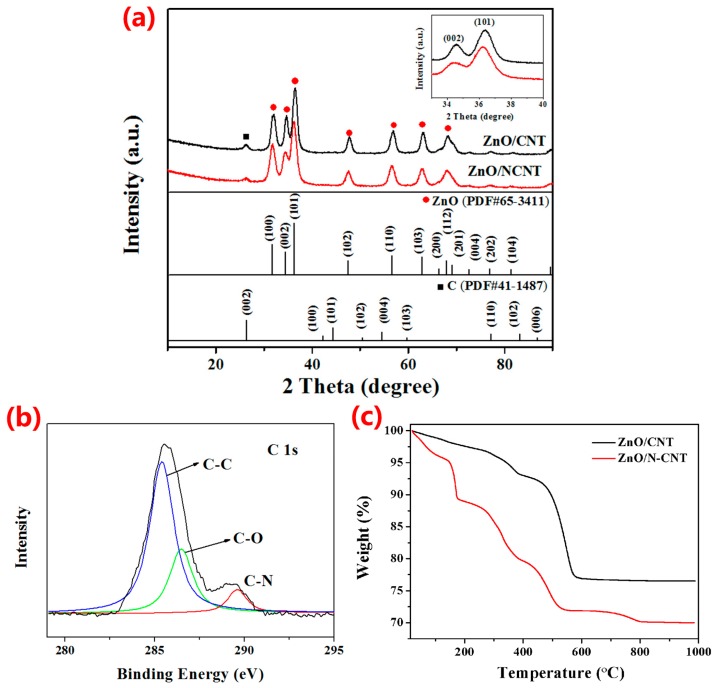
(**a**) XRD patterns of ZnO/NCNT and ZnO/CNT composites; inset: enlarged picture of XRD peaks appearing at 33–40°; (**b**) High-resolution XPS spectrum of C 1s of the ZnO/NCNT composite; (**c**) TG curves of the ZnO/NCNT and ZnO/CNT composites at a heating rate of 10 °C min^−1^ under air.

The XRD patterns of the as-prepared samples are presented in Figure 1a. In the XRD patterns of the ZnO/CNT composite and ZnO/NCNT composite, the location and intensity of the diffraction peaks—which are present at 31.7°, 34.4°, 36.2°, 47.5°, 56.6°, 62.8°, 66.4°, 67.9°, and 69.0°—can be indexed into (100), (002), (101), (102), (110), (103), (200), (112), and (201) crystal planes of pure wurtzite-type hexagonal ZnO (JCPDS No. 65-3411) [29]. Besides, the diffraction peaks are considerably sharp, suggesting the high crystalline ZnO of the composites. A weak peak at 2θ = 26.2° corresponds to the (002) plane of the hexagonal graphite structure (JPDS No. 41-1487), which is similar to that of CNT [30]. It can be seen that there is a slight peak shift of ZnO/NCNT compared with ZnO/CNT (inset of Figure 1a) towards lower angles, which is caused by the N doping on CNT. To demonstrate the variation of the ZnO crystal structure, the lattice spacing was calculated according to Bragg’s formula based on the strongest diffraction peak of (101), and it was found that the lattice fringe spacing changes from 0.247 to 0.252 nm [31]. Compared with the original ZnO crystal, the increased lattice distance of ZnO can provide much more active space and shorten the diffusion distance during the Li-ion insertion/extraction [32]. XPS was used to determine the chemical states of C and N in the ZnO/NCNT. The C 1s region XPS spectrum (Figure 1b) are corresponding to the three broad peaks at 284.2, 285.3, and 288.4 eV, which can be conformed into the C–C, C–O, and C–N species, respectively [33,34]. The nitrogen atomic content in the ZnO/NCNT composite was displayed by XPS analysis as 1.87%, confirming the presence of nitrogen in the composite. It was reported that N doping on CNT can induce chemical adsorption of ZnO nanoparticles on its surface and afford a feasible pathway for Li ions transportation [35]. In order to determine the contents of ZnO in ZnO/CNT and ZnO/NCNT composites, the TG data is collected and illustrated in Figure 1c. Slight weight losses below 150 °C is attributed to dehydration of the starting materials. Major weight losses from about 175 °C to 800 °C are related to the combustion of NCNT and CNT. It can be observed that when the temperature exceeds 800 °C, there are no significant change in the samples weight. Hence, the contents of ZnO in the ZnO/NCNT and ZnO/CNT composites are calculated to be about 75 wt % and 79.5 wt %, respectively.

**Figure 2:**
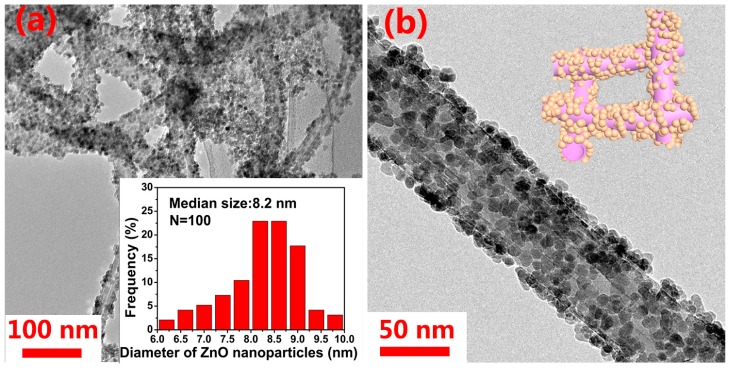

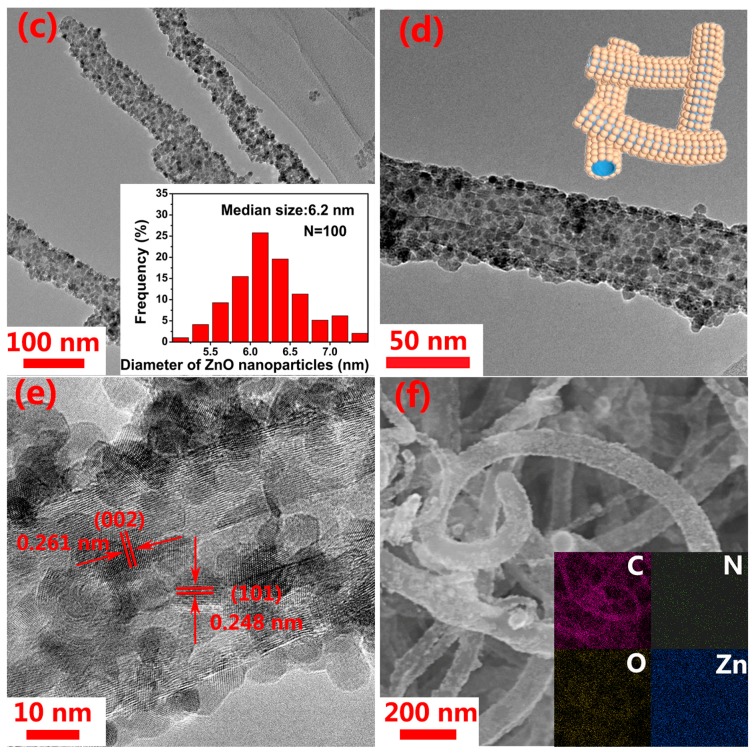
TEM images of (**a**,**b**) ZnO/CNT composite and (**c**,**d**) ZnO/NCNT composites with the particle size distributions (inset); (**e**) HR-TEM image of ZnO/NCNT composite; (**f**) SEM image with the EDX-mapping images (inset) of ZnO/NCNT composite.

To further characterize the morphologies of the ZnO/CNT and ZnO/NCNT composites, TEM was conducted to analyze the size and dispersion of ZnO nanoparticles within the samples. As revealed by the TEM images of ZnO/CNT and ZnO/NCNT composites (Figure 2a–d), one can easily find that the surfaces of both composites are covered by a layer of ZnO nanoparticles. However, the ZnO nanoparticles of ZnO/CNT sample display obvious agglomeration, which may increase the negative effect of the volume expansion during the Li ions intercalation/deintercalation process on cycling performance of the material [36]. It is noticed that the ZnO/NCNT sample shows more uniform dispersion of ZnO nanoparticles with a finer particle size (6.2 nm) than that of ZnO/CNT sample (8.2 nm) (the inset of Figure 2a,c). This phenomenon could be attributed to the alteration of CNT surface properties by nitrogen doping, enhancing its activity and providing more nucleation sites [23]. It could be suggested that the N-doping of CNT, favoring the uniformity of the ZnO nanoparticles dispersion, can ameliorate the electronic conductivity of the composite and its Li-ion storing capability [37]. Crystal lattice fringes spacing of 0.248 and 0.261 nm can be noticed in the HR-TEM image of the ZnO/NCNT composite (Figure 2e), which corresponds to the (101) and (002) plane of ZnO, respectively. Figure 2f displays a SEM image of ZnO/NCNT composite, more clearly the uniform dispersion state of ZnO in the ZnO/NCNT composite can be observed. The EDX mapping (the inset of Figure 2f) indicates that ZnO/NCNT composite contains homogeneously distributed C, N, O, and Zn.

**Figure 3:**
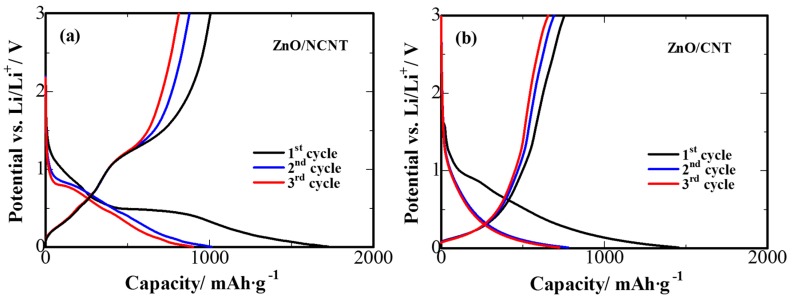
Initial charge/discharge curves of (**a**) ZnO/NCNT and (**b**) ZnO/CNT composite anodes between 0.005 and 3 V at current density of 100 mA·g^−1^.

To evaluate the electrochemical properties of the prepared composites, ZnO/NCNT and ZnO/CNT composites were investigated by galvanostatic cycling in lithium half-cells in potential range of 0.005–3.0 V vs. Li/Li^+^. The initial three charge/discharge curves of the ZnO/NCNT and ZnO/CNT composites at a current density of 100 mA·g^−1^ are exhibited in Figure 3. As shown in Figure 3a, the presence of a prolonged potential plateau at 0.4–0.5 V in the first cycle discharge stage could be ascribed to the decomposition of electrolyte and the formation of solid electrolyte interface (SEI) [38]. The SEI formation causes a low coulombic efficiency and an irreversible capacity loss at the first cycle: the first discharge and charge capacities of the ZnO/NCNT composite are 1727 mAh·g^−1^ and 1005 mAh·g^−1^, respectively, which results in an initial coulombic efficiency of 58%. From the following cycle, the prolonged plateau of SEI formation is not observed and there is only a discharge plateau with an average voltage of about 0.6 V, attributed to the formation of Zn-Li alloy [39]. It can be seen that there is no obvious capacity fading, suggesting a relatively steady state of the lithiation/delithiation process [36]. Meanwhile, a plateau around 0.1 V can be observed in the second and third discharge curves of the ZnO/NCNT composite due to contribution of the carbon matrix into the lithiation process [17]. Compared with ZnO/NCNT composite, ZnO/CNT shows shorter plateaus and lower capacities (Figure 3b). It illustrates that N-doping of CNT plays an important role in maintaining battery capacities. 

**Figure 4:**
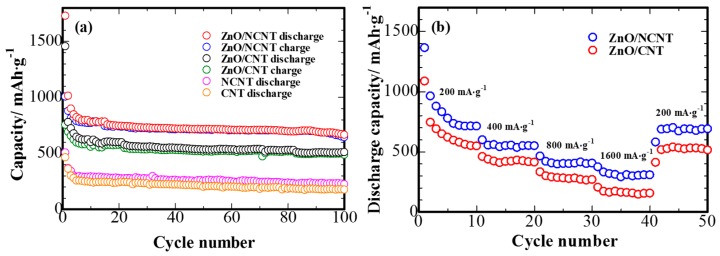
(**a**) Cycling performance of the CNT, NCNT, ZnO/NCNT, and ZnO/CNT anodes at a current density of 100 mA·g^−1^; (**b**) Rate capability of the ZnO/NCNT and ZnO/CNT composite anodes at different current densities from 200 to 1600 mA·g^−1^.

Figure 4a shows the cycling ability data for the ZnO/NCNT composite electrode and its ZnO/CNT counterpart. One can clearly identify superior cycling stability of the ZnO/NCNT composite. The initial reversible discharge capacity of ZnO/NCNT composite at the second cycle is 1013 mAh·g^−1^, and it maintains a discharge capacity of 664 mAh·g^−1^ after the 100th cycle. In the same time, the ZnO/CNT composite suffers from rapid capacity decay, losing about 34% of its second cycle capacity within 100 cycles. For pure NCNT, a reversible capacity of 220 mAh·g^−1^ can be obtained after the 100th cycle, which is slightly larger than that of pure CNT. The comparison of the rate capability for the ZnO/NCNT composite and ZnO/CNT composite is shown in Figure 4b. Apparently, the discharge capacities of ZnO/CNT composite decreases steeply with discharge rate, whereas the ZnO/NCNT composite delivers remarkably higher discharge capacities at relative high energy densities compared with ZnO/CNT: 558 mAh·g^−1^ at a current of 400 mA·g^−1^, 406 mAh·g^−1^ at 800 mA·g^−1^ and 308 mAh·g^−1^ at 1600 mA·g^−1^. Most notably, when the current density switched to 200 mA·g^−1^, the ZnO/NCNT composite could recover its capacity from 308 mAh·g^−1^ at 1600 mA·g^−1^ to 690 mAh·g^−1^ at 200 mA·g^−1^. By contrast, the ZnO/CNT composite displays evidently poor capacity and stability at high current densities.

The improved electrochemical properties of the composite with the nitrogen doped CNT compared with the pristine CNT counterpart could be ascribed to the effect of topological defects on the NCNT [40]: Firstly, the higher concentration of nucleation sites in NCNT favors formation of the smaller size non-agglomerated ZnO particles with more uniform distribution on its surface: this shortens the distances for the lithium ion diffusion during the electrochemical reaction. Secondly, the doping nitrogen atoms make additional contributions due to the capability of offering an extra lone electron pair, enhancing the electrical conductivity of the system compared with the non-doped counterpart [36,41]. The synergetic positive effect of the advantages of use of nitrogen doped CNT allows for preparation of high performance composite ZnO anode for lithium batteries.

## 4. Conclusions

A facile process has been developed for ZnO nanoparticles grown on the surface of NCNT through a one-step sol-gel synthetic technique. The crystal structures and morphology of the ZnO/NCNT and ZnO/CNT were detected by XRD patterns, SEM, and TEM images. Meanwhile, the smaller size and enlarged interlayer spacing of ZnO nanoparticles are dispersed more uniformly on NCNT compared with the CNT counterpart, which was caused by variation of the CNT surface by nitrogen-doping. Therefore, the ZnO/NCNT composite exhibited an enhanced cyclability and improved rate capability as anode for LIBs. Furthermore, the present synthesis strategy provide a low-cost and efficient route to synthesize well-dispersed ZnO nanoparticles deposited on NCNT, which could be extended to preparation of other composite materials.

## References

[1] Armand M., Tarascon J.M. (2008). Building better batteries. Nature.

[2] Zhang Y., Zhao Y., Bakenov Z., Babaa M.R., Konarov A., Ding C., Chen P. (2013). Effect of graphene on sulfur/polyacrylonitrile nanocomposite cathode in high performance lithium/sulfur batteries. J. Electrochem. Soc..

[3] Kundu M., Karunakaran G., Kumari S., Van Minh N., Kolesnikov E., Gorshenkov M.V., Kuznetsov D. (2017). One-pot ultrasonic spray pyrolysis mediated hollow Mg_0.25_Cu_0.25_Zn_0.5_Fe_2_O_4_/NiFe_2_O_4_ nanocomposites: A promising anode material for high-performance lithium-ion battery. J. Alloys Compd..

[4] Kundu M., Karunakaran G., Kolesnikov E., Dmitry A., Gorshenkov M.V., Kuznetsov D. (2017). Hollow (Co_0.62_Fe_1.38_)FeO_4_/NiCo_2_O_4_ nanoboxes with porous shell synthesized via chemical precipitation: A novel form as a high performance lithium ion battery anode. Microporous Mesoporous Mater..

[5] Li H., Li Y., Zhang Y., Zhang C. (2015). Facile synthesis of carbon-coated Fe_3_O_4_ core—Shell nanoparticles as anode materials for lithium-ion batteries. J. Nanopart. Res..

[6] Poizot P., Laruelle S., Grugeon S., Dupont L., Tarascon J.M. (2000). Nano-sized transition-metal oxides as negative-electrode materials for lithium-ion batteries. Nature.

[7] Abbas S.M., Ali S., Ahmad N., Ali N., Abbas S. (2013). Structure and electrochemical performance of ZnO/CNT composite as anode material for lithium-ion batteries. J. Mater. Sci..

[8] Zhou Y.N., Li W.J., Fu Z.W. (2012). Electrochemical reactivity of nanocomposite ZnO-Se for lithium-ion batteries. Electrochim. Acta.

[9] Zhang C.Q., Tu J.P., Huang X.H., Yuan Y.F., Chen X.T., Mao F. (2007). Electrochemical performances of Ni-coated ZnO as an anode material for lithium-ion batteries. J. Solid State Chem..

[10] Huang X.H., Xia X.H., Yuan Y.F., Zhou F. (2011). Porous ZnO nanosheets grown on copper substrates as anodes for lithium ion batteries. Electrochim. Acta.

[11] Wang J., Du N., Zhang H., Yu J., Yang D. (2011). Layer-by-layer assembly synthesis of ZnO/SnO_2_ composite nanowire arrays as high-performance anode for lithium-ion batteries. Mater. Res. Bull..

[12] Laurenti M., Garino N., Porro S., Fontana M., Gerbaldi C. (2015). Zinc oxide nanostructures by chemical vapour deposition as anodes for Li-ion batteries. J. Alloys Compd..

[13] Arora P., White R.E., Doyle M. (1998). Capacity fade mechanisms and side reactions in lithium-ion batteries. J. Electrochem. Soc..

[14] Liu J., Li Y., Ding R., Jiang J., Hu Y., Ji X., Chi Q., Zhu Z., Huang X. (2009). Carbon/ZnO nanorod array electrode with significantly improved lithium storage capability. J. Phys. Chem. C.

[15] Li P., Liu Y., Liu J., Li Z., Wu G., Wu M. (2015). Facile synthesis of ZnO/mesoporous carbon nanocomposites as high-performance anode for lithium-ion battery. Chem. Eng. J..

[16] Hsieh C.T., Lin C.Y., Chen Y.F., Lin J.S. (2013). Synthesis of ZnO@Graphene composites as anode materials for lithium ion batteries. Electrochim. Acta.

[17] Ren Z., Wang Z., Chen C., Wang J., Fu X., Fan C., Qian G. (2014). Preparation of carbon-encapsulated ZnO tetrahedron as an anode material for ultralong cycle life performance lithium-ion batteries. Electrochim. Acta.

[18] Guler M.O., Cetinkaya T., Tocoglu U., Akbulut H. (2014). Electrochemical performance of MWCNT reinforced ZnO anodes for Li-ion batteries. Microelectron. Eng..

[19] Sui J., Zhang C., Hong D., Li J., Cheng Q., Li Z., Cai W. (2012). Facile synthesis of MWCNT-ZnFe_2_O_4_ nanocomposites as anode materials for lithium ion batteries. J. Mater. Chem..

[20] Yang L., Hu J., Dong A., Yang D. (2014). Novel Fe_3_O_4_-CNTs nanocomposite for Li-ion batteries with enhanced electrochemical performance. Electrochim. Acta.

[21] Jeong Y., Lee K., Kim K., Kim S. (2016). Pore-structure-optimized cnt-carbon nanofibers from starch for rechargeable lithium batteries. Materials.

[22] Qin X., Zhang H., Wu J., Chu X., He Y.B., Han C., Miao C., Wang S., Li B., Kang F. (2015). Fe_3_O_4_ nanoparticles encapsulated in electrospun porous carbon fibers with a compact shell as high-performance anode for lithium ion batteries. Carbon.

[23] Liu H., Zhang Y., Li R., Sun X., Désilets S., Abou-Rachid H., Jaidann M., Lussier L.S. (2010). Structural and morphological control of aligned nitrogen-doped carbon nanotubes. Carbon.

[24] Mi R., Liu H., Wang H., Wong K.W., Mei J., Chen Y., Lau W.M., Yan H. (2014). Effects of nitrogen-doped carbon nanotubes on the discharge performance of Li-air batteries. Carbon.

[25] Zhao Y., Yin F., Zhang Y., Zhang C., Mentbayeva A., Umirov N., Xie H., Bakenov Z. (2015). A free-standing sulfur/nitrogen-doped carbon nanotube electrode for high-performance lithium/sulfur batteries. Nanoscale Res. Lett..

[26] Zhao Y., Bakenova Z., Zhang Y., Peng H., Xie H., Bakenov Z. (2015). High performance sulfur/nitrogen-doped graphene cathode for lithium/sulfur batteries. Ionics.

[27] Tao H.C., Yang X.L., Zhang L.L., Ni S.B. (2015). One-step synthesis of nickel sulfide/N-doped graphene composite as anode materials for lithium ion batteries. J. Electroanal. Chem..

[28] Li H., Wei Y., Zhang Y., Zhang C., Wang G., Zhao Y., Yin F., Bakenov Z. (2016). In situ sol-gel synthesis of ultrafine ZnO nanocrystals anchored on graphene as anode material for lithium-ion batteries. Ceram. Int..

[29] Li H., Wei Y., Zhao Y., Zhang Y., Yin F., Zhang C., Bakenov Z. (2016). Simple one-pot synthesis of hexagonal ZnO nanoplates as anode material for lithium-ion batteries. J. Nanomater..

[30] Wang S., Zhou S. (2011). Photodegradation of methyl orange by photocatalyst of CNTs/P-TiO(2) under uv and visible-light irradiation. J. Hazard. Mater..

[31] Hu C., Guo S., Lu G., Fu Y., Liu J., Wei H., Yan X., Wang Y., Guo Z. (2014). Carbon coating and Zn^2+^ doping of magnetite nanorods for enhanced electrochemical energy storage. Electrochim. Acta.

[32] Zhang S., Yu X., Yu H., Chen Y., Gao P., Li C., Zhu C. (2014). Growth of ultrathin MoS_2_ nanosheets with expanded spacing of (002) plane on carbon nanotubes for high-performance sodium-ion battery anodes. ACS Appl. Mater. Interfaces.

[33] Yue H., Wang Q., Shi Z., Ma C., Ding Y., Huo N., Zhang J., Yang S. (2015). Porous hierarchical nitrogen-doped carbon coated ZnFe_2_O_4_ composites as high performance anode materials for lithium ion batteries. Electrochim. Acta.

[34] Zhang T., Zhong B., Yang J.Q., Huang X.X., Wen G. (2015). Boron and nitrogen doped carbon nanotubes/Fe_3_O_4_ composite architectures with microwave absorption property. Ceram. Int..

[35] Song J., Xu T., Gordin M.L., Zhu P., Lv D., Jiang Y.B., Chen Y., Duan Y., Wang D. (2014). Nitrogen-doped mesoporous carbon promoted chemical adsorption of sulfur and fabrication of high-areal-capacity sulfur cathode with exceptional cycling stability for lithium-sulfur batteries. Adv. Funct. Mater..

[36] Cai D., Li D., Wang S., Zhu X., Yang W., Zhang S., Wang H. (2013). High rate capability of TiO_2_/nitrogen-doped graphene nanocomposite as an anode material for lithium-ion batteries. J. Alloys Compd..

[37] Dai J., Wang M., Song M., Li P., Zhang C., Xie A., Shen Y. (2016). A novel synthesis of ZnO/N-doped reduced graphene oxide composite as superior anode material for lithium-ion batteries. Scr. Mater..

[38] Yan J., Wang G., Wang H., Zhang Z., Ruan X., Zhao W., Yun J., Xu M. (2015). Preparation and electrochemical performance of bramble-like ZnO array as anode materials for lithium-ion batteries. J. Nanopart. Res..

[39] Bai Z., Zhang Y., Fan N., Guo C., Tang B. (2014). One-step synthesis of ZnO@C nanospheres and their enhanced performance for lithium-ion batteries. Mater. Lett..

[40] Cai D., Wang S., Lian P., Zhu X., Li D., Yang W., Wang H. (2013). Superhigh capacity and rate capability of high-level nitrogen-doped graphene sheets as anode materials for lithium-ion batteries. Electrochim. Acta.

[41] Du M., Xu C., Sun J., Gao L. (2012). One step synthesis of Fe_2_O_3_/nitrogen-doped graphene composite as anode materials for lithium ion batteries. Electrochim. Acta.

